# The Development Tendency of 3D-Printed Bioceramic Scaffolds for Applications Ranging From Bone Tissue Regeneration to Bone Tumor Therapy

**DOI:** 10.3389/fbioe.2021.754266

**Published:** 2021-12-20

**Authors:** Zhixiang Fang, Jihang Chen, Jiangxia Pan, Guoqiang Liu, Chen Zhao

**Affiliations:** ^1^ Department of Orthopedics, The Second Hospital of Shaoxing, Shaoxing, China; ^2^ Department of Orthopedics, Zhejiang Provincial People’s Hospital, Affiliated People’s Hospital of Hangzhou Medical College, Hangzhou, China; ^3^ Nursing Department, Affiliated Hospital of Shaoxing University, Shaoxing, China

**Keywords:** 3D-printed bioceramic scaffolds, bone regeneration, bone tumor, scaffolds, bone

## Abstract

Three-dimensional (3D) printing concept has been successfully employed in regenerative medicine to achieve individualized therapy due to its benefit of a rapid, accurate, and predictable production process. Traditional biocomposites scaffolds (SCF) are primarily utilised for bone tissue engineering; nevertheless, over the last few years, there has already been a dramatic shift in the applications of bioceramic (BCR) SCF. As a direct consequence, this study focused on the structural, degeneration, permeation, and physiological activity of 3D-printed BCR (3DP-B) SCF with various conformations and work systems (macros, micros, and nanos ranges), as well as their impacts on the mechanical, degeneration, porosity, and physiological activities. In addition, 3DP-B SCF are highlighted in this study for potential uses applied from bone tissue engineering (BTE) to bone tumor treatment. The study focused on significant advances in practical 3DP-B SCF that can be utilized for tumor treatment as well as bone tissue regeneration (BTR). Given the difficulties in treating bone tumors, these operational BCR SCF offer a lot of promise in mending bone defects caused by surgery and killing any remaining tumor cells to accomplish bone tumor treatment. Furthermore, a quick assessment of future developments in this subject was presented. The study not only summarizes recent advances in BCR engineering, but it also proposes a new therapeutic strategy focused on the extension of conventional ceramics’ multifunction to a particular diagnosis.

## Introduction

Traffic accidents, old age, bone tumors, and other causes of bone tissue abnormalities have serious consequences for one’s healthcare as well as living standards (Bădilă et al., 2021) . Large bone abnormalities usually necessitate intermediation treatment in order to recover. Nonetheless, because autogenous bone transplant or autograft is the “gold standard” graft material, supply is limited. As a result, several studies (Wang et al., 2017; Zhu et al., 2017; Lai et al., 2018; Sui et al., 2019) are focusing on developing innovative tissues engineering methodologies for bones tissues regeneration. Scaffold (SCF) are important in bone tissue creation because they provide a three-dimensional (3D) background for cells connection and propagation. Gas frothing (Mathieu et al., 2006), Thaw (Li and Feng, 2005), fibre companionship, particulate/salt leachate (Cao and Kuboyama, 2010; Marques et al., 2017), emulsification (Bohner et al., 2005), phase isolation, and other traditional manufacturing processes are still unable to regulate the porous texture, architectural design, permeability, or interconnectedness of the SCF, and can therefore explicitly and sufficiently enhance cell progression and tissue redevelopment (Butscher et al., 2011). 3D printing (3DP) technique was developed to enterprise and develop SCF with well-ordered chemistries, intended contours, and linked perviousness using computer-aided design (CAD) and computer-aided manufacturing (CAM) to address the limitations of traditional manufacturing techniques (Bose et al., 2013; Brunello et al., 2016; Guvendiren et al., 2016; Bădilă et al., 2021).

A standard scaffold for bone tissue engineering (BTE) should mimic the shape and physiological activities of typical bone tissue in based on its chemical mixtures, rigid hierarchy, and features. Ceramic-based SCF [e.g., Ca-salts-ceramics, Ca-Si (CS) ceramics, and bioactive-glasses (BGs)] are drawing considerable interest to be used in BTE due to their resemblance to indigenous bone chemical compositions, bio—compatibility, wettability, biological activities, osteoinduction, and printability (Woodard et al., 2007). Bioceramic (BCR) frameworks with such a hierarchical system that comprises macroscopic, microscopic, and nanoscale structures have also been created. SCF have indeed been intended with various macrostructures (e.g., pore size, permeability, and pore connectivity) to enable tissue expanding and appropriate transportation of nutrients, oxygen, waste, and growth factors, and to also encourage effective tissue regeneration cell development from the fringes to the interior of the SCF(Bose et al., 2013). SCF with submicron and nanoscale structures have more contact area and ruggedness, which encourages osteoblast adhesion to the scaffold surfaces (Park et al., 2007).

**FIGURE 1:**
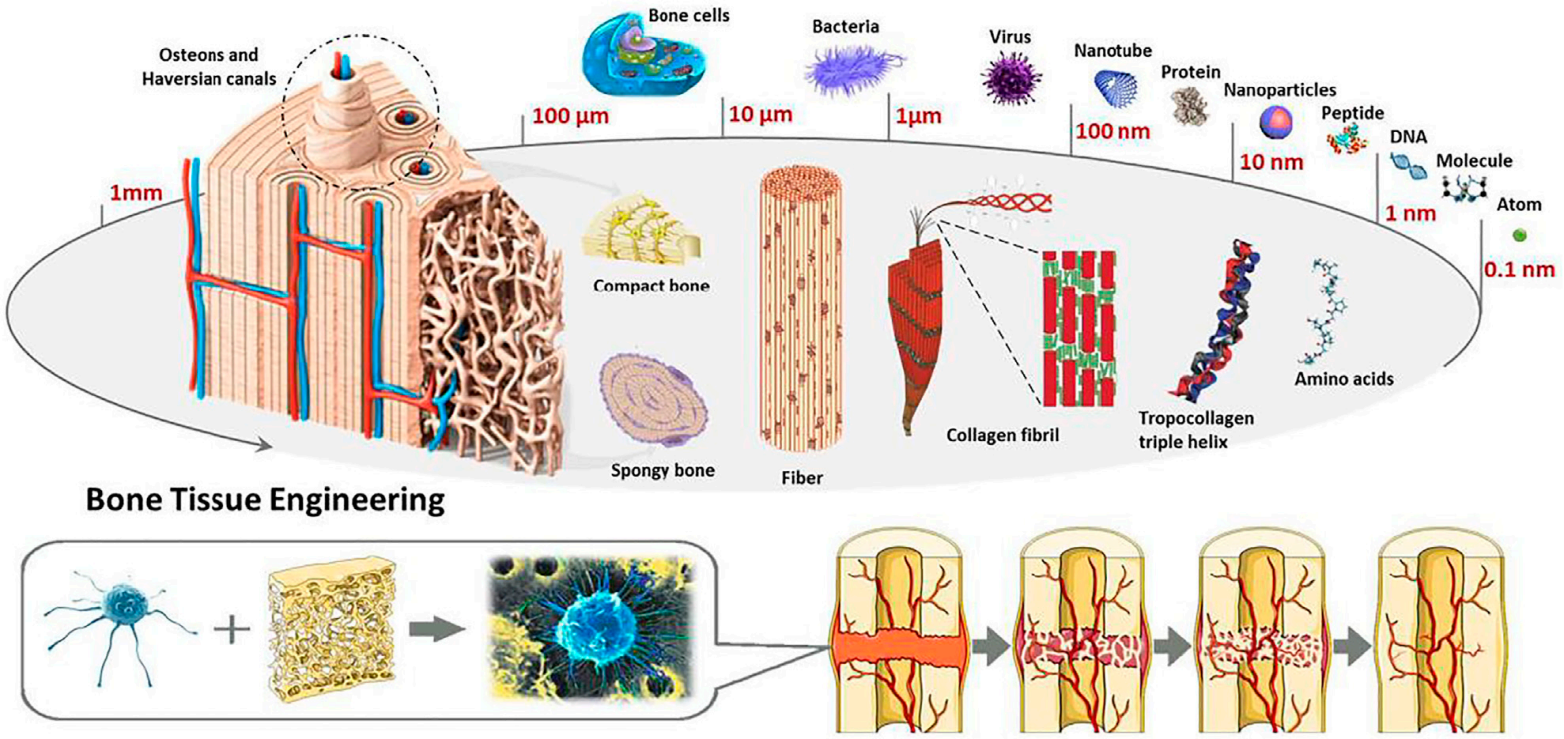
Microstructure and composition based bone tissue engineering. Reproduced with permission from (Hao et al., 2017).

Few studies of 3D-printed ceramics SCF about bones tissues engineering have been published (Nowicki et al., 2017), summarizing just 3DP technologies and various types of ceramic-SCF. The goal of current review, on the other hand, is to sketch out the development trend of 3DP-B SCF for applications ranges from bones tissues regeneration to bone tumor treatment. The prior step was to demonstrate 3DP-B SCF with various configurations and structural organization topologies (macros-, micros-, and nanos-scales). Conventionally, 3DP-B SCF were manufactured and primarily used to regenerate bone tissue. This study, on the other hand, emphases on current progresses in functional 3DP-B SCF that could be utilized for both cancer therapy and BTE. Such serviceable BCR SCF offer a lot of promise in terms of fixing surgically produced bone defects and killing any remaining tumor cells in order to attain bone tumor treatment. Lastly, a quick assessment of forthcoming developments in this topic is presented (Zhang et al., 2017a). Bones, like natural nanocomposites in the human body, have multi-layer architectures with complicated compositions, as seen in Figure 1.

## 3-Dimensional Printing Manufacturing Techniques Benefits

Traditional fabrication procedures such as gas flocculation, fibre bonding, freeze dryness, phases isolation, and particle rinsing were used to create most SCF at first (Lu et al., 2021) . Conventional strategies, but at the other hand, are unable to regulate the SCF’ porosity structure, morphology, permeability, or interconnectedness, and the resulting SCF cannot be specifically tailored to facilitate angiogenesis and tissue regeneration (Li et al., 2014). Conventional manufactured products for the manufacturing of BCR SCF have been extensively used to overcome the limitations of 3DP techniques based on CAD and CAM (Jin Woo and Dong-Woo, 2015). Professor Sachs of MIT (Massachusetts Institute of Technology) was the first to create the term 3D printing innovation, which really is a form of rapid prototyping technology (Liu et al., 2013). There seem to be a variety of 3DP techniques, which may be classified depending on the method of construction. The methods utilized are stereolithography (SLA), selective laser sintering (SLS), fused deposition modeling (FDM), and binder-based 3DP3DP (Hwa et al., 2017). SLA SCF are created by splitting a based model sliced by sliced first from head to tail, then solidifying it by using UV laser beam. Instead of utilizing a UV laser, SLS uses sintered approach to produce materials. In SLS, the laser is utilized to polymerize the granules and fuse them collectively (Yves-Christian et al., 2010). SCF are constructed in FDM by layering filaments on the workstation. The filaments is dissolved and ejected from a throttling valve (Luo et al., 2013), that is typically made of ceramics plus binders. In 3DP, ceramics are diluted with water binders to promote binding among powder particles and SCF, allowing them to form their desired forms (Pham and Gault, 1998).

The advantages of fabricating bio-ceramic SCF using the 3DP approach are as follows: 1) SCF’ inner and exterior architectures, such as pore form, perviousness, and interconnectedness, could be precisely manipulated to achieve extraordinary organizational complication, elasticity, and patient-particular burdens (Jin Woo and Dong-Woo, 2015). 2) To restore bone tissue, optimal 3DP SCF deliver a growth-directing construction for cell migration and proliferation (Vaezi et al., 2013). 3) 3DP enables rapid construction while reducing experimental mistakes (Vaezi et al., 2013).

### Benefits of 3-Dimensional Printing-B Scaffolds

Owing to their resemblance to the synthetic formulation, impact strength, wettability, bioactivity, bio - compatibility, osteoconductivity, and possible osteoinduction of the bone, bio-ceramics have become the subject of intense research for years (Woodard et al., 2007). They promote wound healing by modifying the *in vivo* conditions. The exterior of biocomposites SCF absorbed osteoinductive chemicals and/or ions [e.g., calcium (Ca), phosphate (PHPH), and silicate (Si)] ions from the surroundings, encouraging MSC growth (Asa’ad et al., 2016). 3DP-B SCF should have the following characteristics: 1) Porous structure which can be regulated and is consistent, such as pores, size distribution, direction, and interconnectedness. Such a permeable assembly is advantageous for nutrient delivery and cell migration into SCF. 2) Sufficient machine-driven asset that can be accurately regulated to fulfil a variety of clinic requirements. 3) Bio-compatibility and bio-degradability, which ensures that SCF decay into harmless compounds with minimum inflammatory reactions. 4) Bioactivity, which results in the development of biochemical linkages between SCF and physiological system by generating specific bio-chemical responses at the boundary between materials and biological tissues. Furthermore, 3DP SCF can be bifunctional, allowing them to be employed for both tumor treatment and bone regeneration.

The use of mesoporous bioactive glasses (MBGs) to fabricate scaffolds *via* 3D printing has been identified as having significant promise for the creation of bioactive synthetic bone replacements. The obvious advantages of additive manufacturing make it an attractive strategy for circumventing the most common limitations of traditional fabrication processes for glass and ceramic scaffolds, such as poor reproducibility and control over the final 3D structure, as well as low mechanical strength. Additionally, the highly tailorable textural characteristics of mesoporous materials were discovered to be critical in the construction of multifunctional systems capable of concurrently supporting bone repair and therapeutic action via local delivery of medicines and/or biologically active ions (Baino and Fiume, 2020). Table 1 listed some of the key studies that reported MBGs along with the details.

**TABLE 1:** Selected studies using 3D-printed mesoporous bioactive glasses material, their parameters, manufacturing and other structural details.

Material	Binding agent	Mesopore SDA	BET surface area (m^2^/g)	Dispensing pressure (kPa)	Size (µm)	Reference
SiO_2_-CaO-P_2_O_5_ MBG/PCL composite	poly(caprolactone)	F-127	520	Not reported	190	Yun et al. (2011)
SiO_2_-P_2_O_5_ MBG	methyl cellulose	F-127	152–310	Adapted during printing	400	García et al. (2011)
CSH/SiO_2_-CaO-P_2_O_5_ MBG	poly(caprolactone)	P123	4.5–12.8	220–360	350	Qi et al. (2017)
Carboxylic-modified SiO_2_-CaO-P_2_O_5_ MBG	poly(3-hydroxybutyrate-co-3-hydroxyhexanoate)	P123	63–330	Not reported	250	Zhu et al. (2015)
SiO_2_-CaO-P_2_O_5_ MBG/alginate composite	Alginate	P123	Not reported	180–250	344–415	Luo et al. (2013)

## 3-Dimensional Printing-B Scafflold for Biomedical Applications Together in Variety of Components

Bio-ceramics for bone tissue manufacturing include Ca-PHPH ceramics, CS ceramics, and BGs, among others. The utilisation of 3DP-B SCF in BTRis determined by their printable, mechanical, and biological characteristics. Lewis et al. have published numerous papers on the construction of complicated 3D-printed SCF with improved performance. They also gave a thorough overview of the various characteristics and parameters of 3DP methods on ceramic constructions.

### Calcium-(poly (3-hydroxybutyrate- co-3-hydroxyhexanoate) Bioceramic Scaffold

Different reports have been revealed that some Ca-PHPH (poly (3-hydroxybutyrate-co-3-hydroxyhexanoate) ceramics are osteoinductive in the absent of supplementation (Samavedi et al., 2013). Moreover, the chemical nature and charges of calcium PHPH ceramics impact their osteoinduction capacity, that might change permeation rate and, as a consequence, endorse angiogenesis via cell–extracellular matrix interactions (Samavedi et al., 2013). Calcium PHPH ceramics’ structural quality in the context roadblock to their broad clinical application. As little more than a consequence, Ca-PHPH ceramics are used as non-load-bearing implantation in middle ear operations and to replace bone defects in the oral cavity and bones, as well as as a coating for dentistry and orthopaedic metallic implants. The fracture toughness of Ca-PHPH ceramics is related to having primary ionic bonding. To enhance biocompatibility and good mechanical of calcium PHPH-based SCF, Awad et al. developed a special equation comprising 8.75 wt percent phosphoric acid, and then manufactured a Ca PHPH-based scaffolding at low temp. Such combinations improved the scaffold’s cytocompatibility and bone regeneration while also enhancing its mechanical properties in a significantly sized femur fracture (Inzana et al., 2014).

Hydroxyapatite (HA) is a kind of calcium PHPH. HA (Ca10(PO4)6(OH)2) is an utmost studied Ca PHPH bio-ceramics in bone tissues engineering based on the chemical arrangement, which is comparable to that of the primary bone components, resulting in beneficial effects on osteoblast adhesion and proliferation (Kumar et al., 2019). This may be used to optimize the material behavior in biological settings by developing HA with a particular surface structure and electric charge (Salinas et al., 2013). Pure HA, on the other hand, has a slow degradation rate, low mechanical strength, and low fracture toughness, which prevents full bone regeneration and may raise the danger of contamination (Lu et al., 2002; Ni et al., 2008). As a result, HA was mixed with other alignments such as zirconium oxide (Rapacz-Kmita et al., 2006), carbon fibre (Suping et al., 2004), and Al2O3 (Lee et al., 2007) in order to increase mechanical qualities and boost HA’s capabilities for bone tissue regeneration. However, bioinert materials such as ZrO2, carbon fibre, and Al2O3 greatly decrease the bioactivity of HA (Inagaki and Kameyama, 2007). Furthermore, when natural polymers with quicker kinetics were added, a nanocomposite scaffold having a low rate of decomposition was created. Polylactide-co-glycolide acid (PLGA) is one of the most extensively used polymeric biomaterials, with excellent biodegradability and biocompatibility. As a result, Yang et al. used a 3DP method to construct porous PLGA/HA SCF (Yang et al., 2016).

Tricalcium PHPH (TCP) is another typical Ca-PHPH ceramic, because to its quick breakdown degree and capability to establish a strong bone–calcium PHPH connection. Certain biomimetic implants, including HA, have indeed been proven to deteriorate more rapidly as TCP ceramics. Porosity, stiffness, and tenacity (the min particle size and parting space of the struts, that could be exactly regulated by a 3DP) of 3DP TCP-SCF are all influenced by particle diameter, depowering effectiveness, adhesive droplet size, and scaffolding shape (Tarafder et al., 2015). Most favoured type of TCP scaffolding is b-TCP and for its structural chemical resistance durability. Following American physician Fred Houdlette Albee’s original effort in 1920 to implant b-TCP as a synthetic material to mend operationally induced cracks in rabbit’s skeletons, b-TCP ceramic gained increasing interest due to its excellent biocompatibility and osteogenesis (Eliaz and Metoki, 2017). Peer groups studies demonstrate that BCP ceramics have a reduced biodegradation, higher biocompatibility, and improved bone regeneration ability than pure HA and pure b-TCP SCF(Yamada et al., 1997; Ryu et al., 2004; Wu et al., 2011).

### Calcium-Silicon Bioceramic Scaffold

CS (CaSiO3) ceramics, a form of unique bioactive substance, have been inspected for bone regeneration (De Aza et al., 2000). These composites can stimulate the development of a carbonated HA coating by immersing in simulated bodily fluid (SBF), which can generate a strong chemical link between the biopolymer and the bone tissue along the biomaterial (Kokubo and Takadama, 2006; Xu et al., 2008). Zhao et al. (2005) developed a very consistent CS SCF with a pore-controlled assembly and good mechanical features using the 3DP technique. The 3DP CS SCF had such a reasonably easy manufacturing process, outstanding flexural modulus, satisfying apatite-mineralization capacity in SBF, and an elevated rejuvenating of bone deficiencies, asserting that they would have huge potential.

### Bioactive Glasses

Hench was the one who discovered BGs. Na2O, CaO, SiO2, and P2O5 are the major components of bioactive glasses (BGs). BGs offer a lot of promise in terms of repairing and regenerating bone deficiencies. BGs have osteoconductiveness and oseteoproductiveness, which improves progenitor cell proliferation and differentiation (Rainer et al., 2008). Furthermore, because of its ability to induce angiogenesis in response to the action of vascular endothelial growth factor (VEGF), BG is a viable substitute to conventional scaffolding biomaterials (Kentleach et al., 2006). Preceding research has shown that BGs may be used as sintering aids to improve BCRs’ mechanical and biotic properties. Winkel et al. (2012) sintered a BG-reinforced HA amalgamated SCF with an orientated pore at a truncated temperature. Lin et al. (Suwanprateeb et al., 2009) produced 45S5 BG-reinforced wollastonite permeable ceramics with a compressive asset of 109 MPa.

### Different Doped Elements

Biotic apatite’s, a chemical component of bone, contain a definite quantity of Zn++, Sr++, Mn++, and Mg++ in addition to HA. These ions have their own roles, and by gently releasing these therapeutic ions, they stimulate osteogenesis and angiogenesis.

The integration of Zn++ in HA ceramics increases the dissociation property, encourages osteoblastic activity from bone marrow stromal cells *in vitro*, and obviously boosts new bone formation *in vivo* (Li et al., 2009) by repressing osteoclast differentiation by antagonizing NF-jB activation motivated by TNFα, an inhibitor of bone formation *in vitro* and *in vivo* (Li et al., 2009).

Manganese (Mn), an abundant mineral component for regular human metabolism (e.g., biosynthesis in bone tissues), has many benefits as a dopant factor for tissue engineering (Mondal et al., 2010), which include excitation of angiogenesis process (Armulik et al., 2000), augmentation of the bioactivity of cartilage oligomeric matrix protein (Liu et al., 2018), and advancement of osteogenic activity *in vitro* (Srivastava et al., 2012). Mn ions might even improve the quantity of BMSC repositories in the bone tissue, that are otherwise stem cells which may grow into osteoblasts, chondrocytes, and adipocytes (Wang et al., 2014). The development of new vascular system in the interior regions of the transplanted SCF is related with tissue engineering and a modest rate of vasculature (Santos and Reis, 2010).

#### Phosphocalcic Cement

Historically, Legeros et al. (1982) was the first to suggest biomaterial phosphocalcic cement. Since then a range of preparations have been produced, analyzed and commercialized (Mirtchi et al., 1989). Cements are a class of biomaterials distinguished through their galenic composition. These are the alternative to high thermostable ceramics. Therefore they can be assumed that the cement is made up of pulverized liquid and solid phases, when combined in the correct ratios, is fixed and hardened. Different phosphocalcium cements, currently employed in orthopaedic and dental-surgery. Mineral hydraulic (MH) cements could be distinguished from polymeric cements by containing mineral fillers). They contain two main categories: the former is apathetic, like CaCO3 or TTCP, and second is a brushite, corresponding to H_3_PO_4_ or DCPD (Mestres and Ginebra, 2011; Tamimi et al., 2012). Some phosphocalcic cements are put on the market, some cements and their formulations are shown in Table 1 for the usage of these bio-ceramics is confined by their low mechanical attributes. Table 2 list various commercially available phosphocalcic cement.

**TABLE 2:** Examples of commercially available phosphocalcic cements (Monma, 1988).

Name of cement	Manufacturer	Solid-phase composition	Liquid-phase composition	S/L	
α—BSM (Biobon, Embarc)	ETEX	ACP + DCPD	Physiological serum	0.67 (g/cm^3^)	
VitalOs	CalciOS	1: βTCP + Na 2H2P2O7	1: water		
		2: MCPM + CaSO 4, 2H2O	2: water + H3PO4		
Eurobone	Kasios	βTCP + Na4P2O7	water + H2SO4		
Bonesource	Stryker—Leibinger Corporation	TTCP + DCPA	Na	0.25 (g/cm^3^)	
			2HPO4 +		
			NaH		
			2PO4		
Calcibon	Biomet	αTCP + DCPA + CaCO3 + PHA	Na2SO4		
ChronOS Synthes		βTCP + MCPM + MgHPO4 + MgSO4	Na2H2P2O7 Hyaluronate de Sodium		
Cerapaste (Primafix)	NGK Spark Plug	TTCP + DCPA	Sodium		

## 3-DPrinting-BScaffold With Varied Macro/Microstructures for Bone Tissue Engineering

**FIGURE 2:**
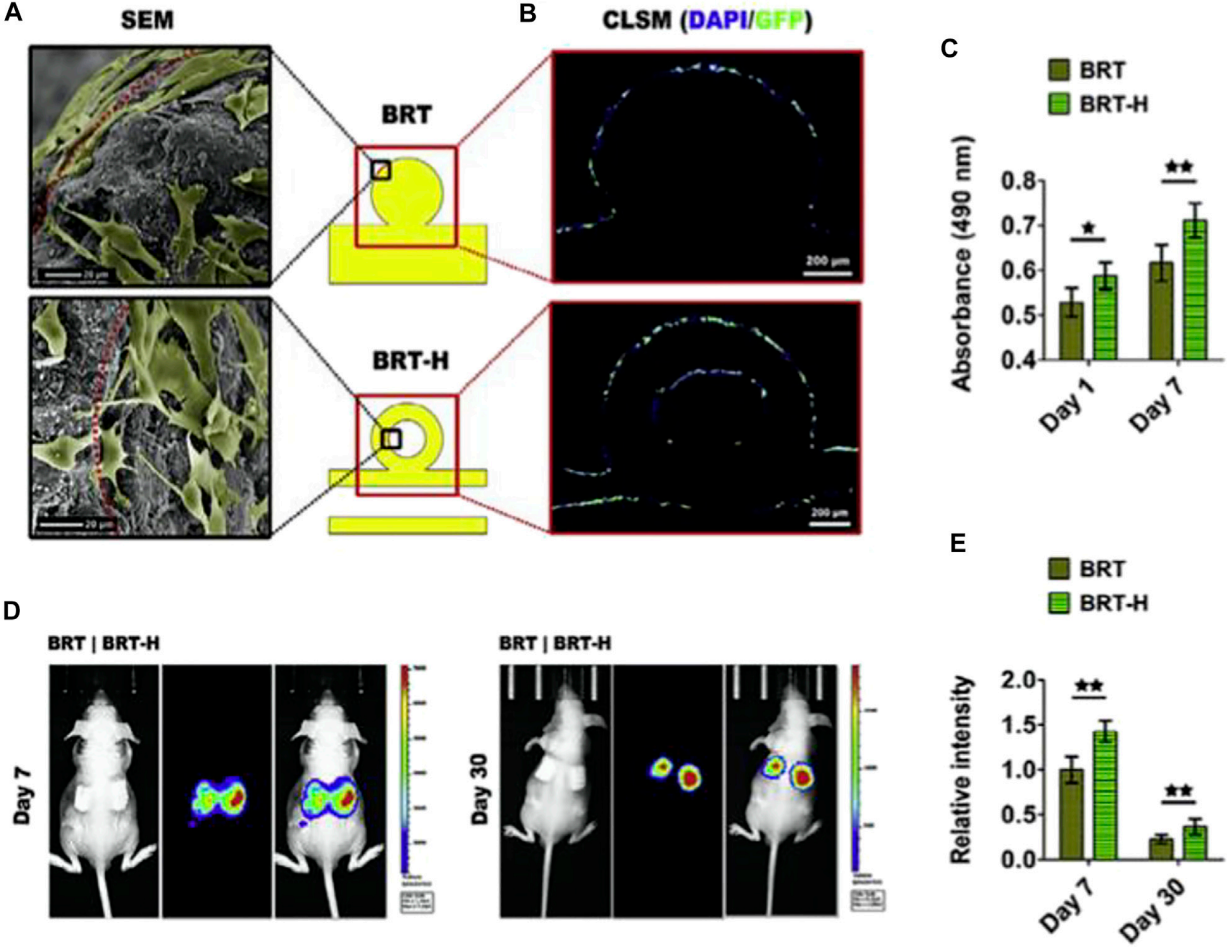
The impact of hollow tubes on the delivery of stem cells. **(A)** Scanning electron microscope images of adhering rBMSCs on the scaffold surface. In the hollow channels of the BRT-H scaffolds, cells may be seen. **(B)** After 7 days of *in vitro* cultivation, the cells in the hollow channels were still alive. **(C)** Cell viability of rBMSCs planted in scaffolds on day 1 and day 7. **(D)** Mice were implanted with scaffolds containing luciferase-labeled BMSCs, which were identified using *in vivo* fluorescence imaging. **(E)** Relative fluorescence intensity statistical findings. Reproduced with permission from (Zhang et al., 2017b).

Among the most challenging problems encountered in area of biomedical applications is the fabrication of hierarchical porous SCF that imitate the conformational shape and features of actual bone. To demonstrate features, nutrition delivery, and cell–matrix interactions, the architecture must be developed at the macros, micros, and nanos dimensions (Figure 2). Macrostructure is important in BTE because it is closely linked to the degree of bone ingrowth. Porous structure, pore diameter, pore shape, and pore interconnectivity, in particularly, have a significant influence on scaffold function, cell penetration, and adhesion to scaffold macropores, all of which play a role in osseointegration.

### The Influence of Macrostructure on Physico-chemical Assets

BCR SCF are being reported extensively in bone regeneration due to their resemblence to bone inorganic compounds, bioactivity, bio - compatibility, osteoinduction, and probable osteoinductivity. Nevertheless, in a number of cases, their mechanical properties limited their application. As a consequence, scientists attempted to modify the porosities of BCR SCF to generate mechanical properties which suited the requirements of the ultimate implementation. Roohani-Esfahani et al. developed Sr-HT Gahnite, a BG ceramic with a novel triphasic microstructure comprising of strontium (Sr)-doped hardystonite (Ca2ZnSi2O7, HT) grains, clustering of submicron gahnite (ZnAl2O4) crystals, and a glass phase. In attempt to construct Sr-HT Gahnite 3DP SCF with improved tensile strength, they employed a range of pore geometrie with a range of porosities (Roohani-Esfahani et al., 2016).

In comparison to another pore types, SCF with hexagonal designs displayed the peak tensile strength anywhere at particular porosity, leading in a substantially anisotropy morphology and enhanced oad transmission. Because as average diameter grew, the compressive strength of scaffolding with equal pore geometries decreased (450, 550, 900, and 1,200 lm). Sr-HT Gahnite SCF (hexagonal, 50% porosity) have a compressive strength of 180 MPa, demonstrating that they might be utilised to repair load-bearing bone abnormalities (Roohani-Esfahani et al., 2016).

### Influence of Macrostructure on Biological Features

**FIGURE 3:**
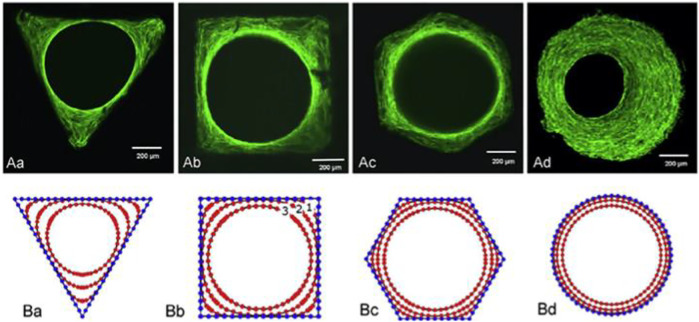
Tissue development in channels with various cross-sections: triangular, square, hexagonal, and circular. **(A)** Phalloidin-FITC stained actin stress fibres for various geometries. **(B)** A computational simulation of tissue development depicting several tissue evolutions. Reproduced with permission from (Gariboldi and Best, 2015).

The geometry of macropores can influence cell network creation and tissues implantation (Figure 3) (Gariboldi and Best, 2015). Cells want a modulus of elasticity that is much larger than their own radius of curvature (Zadpoor, 2015). In reaction to curvature, murine osteoblast-like cells migrated, proliferated, or differentiated, ensuing the early tissue creation at the angles [96]. High curvature induces mechanical stresses in cells, as evidenced by the formation of actin stress fibres at the tissue–fluid interface, which drive future tissue growth.

Wu et al. employed a customized 3DP methodology to a multichannel structure, that addresses the constraints of the standard 3DP method (Feng et al., 2017). They were influenced by the root of the genuine lotus plant. These bioinspired SCF’ physicochemical properties may be tweaked. The lotus root-like form of the biomimetic materials aids nutrition transport and oxygen distribution in the inner part of the scaffold. Furthermore, by encouraging circulatory system and new bone tissues to grow into the inner portion of the biomimetic materials, this lotus root-like structure may reliably and efficiently promote bone defect healing. The angiogenic and osteogenic stimulatory ability of conventional 3DP materials is lower than that of lotus root-like biomimetic materials.

The use of innovative HSP BCR SCF with customized ceramic particles and multioriented hollow channel designs to promote bone regeneration yielded the following findings (Luo et al., 2015). Initially, hollow channel designs induced fast Si ion migration from of the SCF. Secondly, the increased degradation of the HSP scaffolding allowed for greater area for the establishment of new bone tissue. Thirdly, the hollow tube assisted bone formation by acting as a conduit for oxygen and nutrition supply as well as cell movement. In contrast to potential osteogenic effects, the bioactive ions generated by hollow-pipe-packed Si BCR SCF can endorse angiogenesis by promoting endothelial cell migration. Further significantly, the hollow pipes seem to enable the infiltrating of host blood arteries into the hollow channels, and also the transfer of stem cells and growth hormones, both of which help tissue regeneration (Zhang et al., 2017b). (Figure 2).

## Effect of Micro/nanostructures on Bone Tissue Regeneration

3DP SCF offer greater interconnectedness, ordered crystallinity, and tensile stability than SCF made using conventional techniques, making them appropriate for bone tissue production. Cell leakage from the large hole in 3DP-BSCF is, however, one of their major disadvantages. The creation of hierarchically 3DP-BSCF was detailed in order to imitate the structure of nature. Cell expansions can be provided with anchored sites by the hierarchial porous structure of 3DP SCF, enabling them to multiply as well as permeate the biological material. In addition, the osteogenic activity and bioactivity of hierarchically porous materials is much improved. Several methods for producing hierarchically porous 3DP SCF have been endeavored. The following are a few instances of common questions.

### Freeze-Drying Technique

Using 3DP technologies and the thawing process, Xu et al. constructed porous BCR-silk composite SCF with hierarchical pore topologies, that were helpful for osteogenesis. By freeze-drying hierarchial composite SCF, ordered macropores of BCR SCF and micropores of silk networks were produced. The composite SCF have ordered hierarchical pore structures, excellent apatite-mineralization capabilities, and mechanical characteristics with a compressive strength of 25 MPa. The foliage of sheet-shaped polymeric SCF generated mesopores, and the highly porous functions as a net bag to restrict cell leaking from the mesopores of ceramic scaffolding during cell growth. A particular range of enhanced cell density, that is advantageous to cell development, proliferation, and differentiation, aids intercellular interconnections and paracrine communication (Xu et al., 2015).

### Self-Assembly Method

**FIGURE 4:**
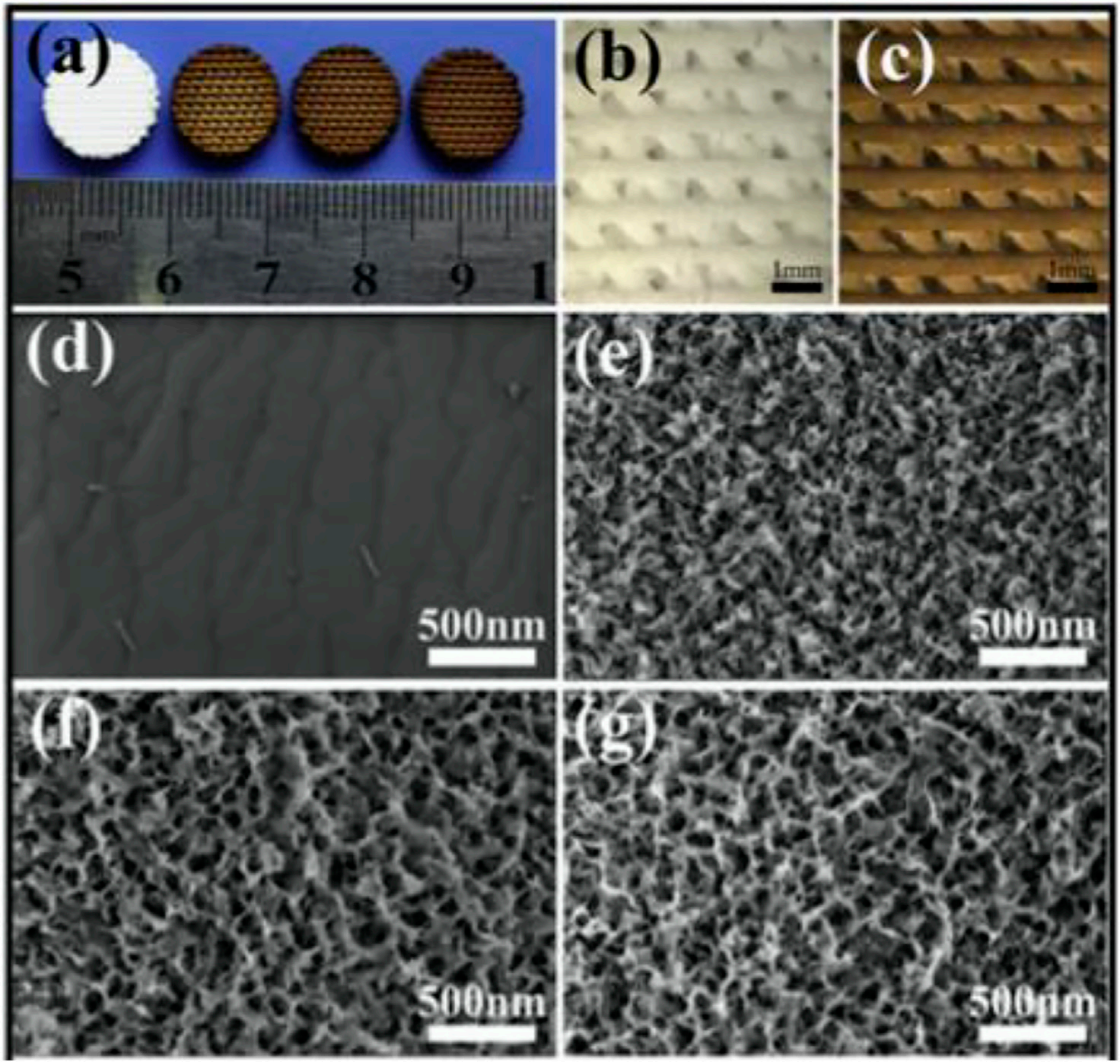
**(A)** 3D-printed pure bioceramics (BC), DOPA-BC (2 mg/ml), DOPA-BC (4 mg/ml), DOPA-BC (6 mg/ml) scaffolds; **(B)** Optical photos of pure BC (b) and 4 mg/ml DOPA-BC **(C)** scaffolds on top view; **(G)** SEM images of pure BC **(D)** and 2 mg/ml DOPA-BC **(E)**, 4 mg/ml. Reproduced with permission from (Ma et al., 2016a).

A hierarchy BCR scaffolding with regulated mesopores and mussel-inspired exterior nanoparticles was produced using a purely self approach (Xu et al., 2013). The self-assembling Ca-P/polydopamine complex nano-layer enhanced the surface coarseness and wettability of amalgamated SCF (Figure 4). On the one side, the presence of OH–and NH2– in the Ca-P/polydopamine composite nanolayer may help increase the surface hydrophilicity of composite BCRs. But at the other side, pure b-TCP BCR SCF have a thick surface. On the other contrary, the surface of composite SCF with a Ca-P/polydopamine nanolayer is loose, which increases wettability. In contrast, calcium PHPH chelated polydopamine nanolayers significantly improved adhesion strength, cell proliferation, bone-related gene expression, ALP activity, and osteogenic protein synthesis in BMSCs when comparing to pure TCP SCF. The boosted biotic retort of cells is largely due to the hybrid nanostructures and bioactive functional groups. Through enhancing surface quality and furthermore affecting serum protein adherence, the nanostructure may increase cell proliferation and differentiation, hence changing substrate–cell interaction and thus enhancing cell proliferation and differentiation (Ma et al., 2016a).

### Spin Coating Technique

Zhang et al. created MBG-modified b-TCP SCF with a functioning MBG nanolayer and a hierarchy pore structure evenly covered on the scaffolding columns using 3DP and spin coating methods (Zhang et al., 2015). Micro defects and grain boundaries on the strut faces of bTCP SCF were plugged using MBG nanolayers, possibly increasing the composite SCF′ compressive strength. Luo et al. created a hierarchy SCF comprised of an MBG/alginate composites with size-controlled mesopores and very well planned nanochannels for drug delivery. Within that system, the MBG particulates enhanced the mechanical properties and apatitemineralization capability of alginate. Alginate is used for a variety of reasons, including its high biocompatibility, biodegradation, and capacity to act as a drug delivery system (Luo et al., 2012).

### Hydration Process

A innovative scaffolding incorporating calcium sulphate hemihydrate (CSH) and highly porous calcium Si (MCS) for BTEwas formed utilizing 3DP and hydration processes, with CSH illustrating functionality in raising compressive strength, prohibiting rapid pH increases, and balancing biodegradation. Moreover, the MCS component may load dexamethasone into particular regions and release it at a slow rate. BTE might benefit from the SCF (Pei et al., 2017) .

### Nano-Stereolithography

“Cha et al. utilized the nanostereolithography (NSTL) technology to integrate micro patterns on the scaffolding. They examined the effects of micropillar and microridge patterns on cell adhesion, proliferation, and osteogenic differentiation on 3Dprinted SCF. They observed that SCF with micropatterns seemed to improve cell adherence when compared to non-patterned SCF. Investigators also perceived that bone-related gene expression, such as Runx2 and ALP, was much greater in micro patterns (micro-pillar and micro-ridge) than in non-patterned SCF(Cha et al., 2012)”.

## 3-Dimensional Printing-B Scaffold for Bone Tumor Therapy

**FIGURE 5:**
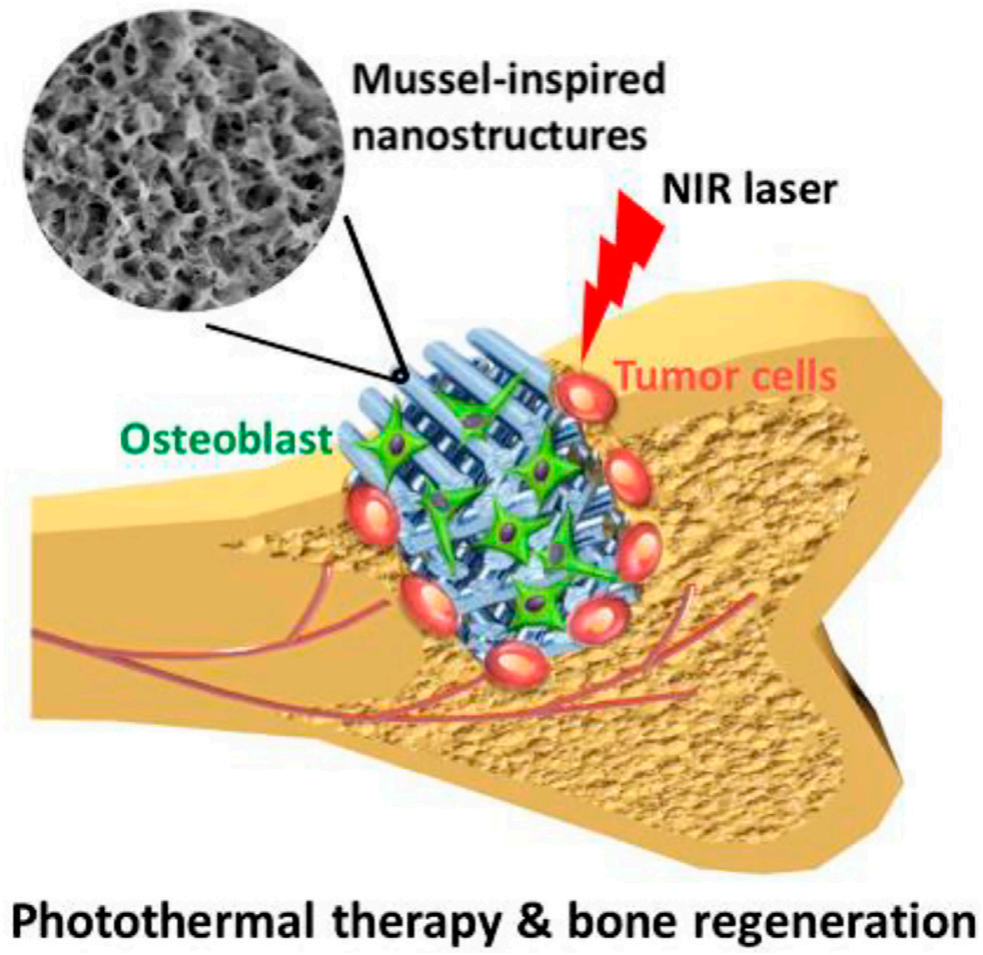
Bifunctional scaffold with nanolayer developed with 3D Printing and dopamine modification. Reproduced with permission from (Ma et al., 2016a).

A significant integer of 3DP-BSCF with predetermined configuration and construction have been created and shown to have good physicochemical and biotic presentation, suggesting that they might be used in BTE. In addition to its use in bone tissue engineering, new functional BCR SCF have been shown to have the capacity to treat tumors and regenerate bone tissue at the same time. As a result, these practical SCF may be used to mend bone defects caused by surgery while also killing any remaining tumor cells, achieving the goal of bone tumor treatment (Figure 5). Unlike chemo/radiotherapy, photothermal/magnetothermal therapy has minimal adverse effects and may selectively and efficiently ablate tumor cells without harming healthy tissue. Permanent proteomic degradation, cellular membranes degradation, and delayed advanced apoptosis are all caused by high temperatures generated by functional SCF. As a result, photothermal or magnetothermal agents can be used locally with functional SCF that have good photothermal or magnetothermal performance. Furthermore, these functional BCR SCF have good biocompatibility, encourage bone MSC migration, attachment, proliferation, and differentiation, and indorse fresh bone creation *in vivo*. Some researchers have recently concentrated on the development of such functional 3DP SCF for use in bone tumour treatment.

GZ-modified BCR scaffolding with good photothermic upshot was created by Wu et al. (Ma et al., 2016b). In mice, the unique photothermal impact of useful SCF may be regulated to efficiently ablate tumor cells and prevent tumor development. It has been demonstrated that high temperature produced by functional SCF significantly reduced tumor cell growth while significantly promoting tumor cell apoptosis. Furthermore, due to the bioactive groups and protein absorbance of GO, functional SCF enhanced the osteogenic differentiation of rabbit bone MSCs when compared to pure BCR SCF.

**FIGURE 6:**
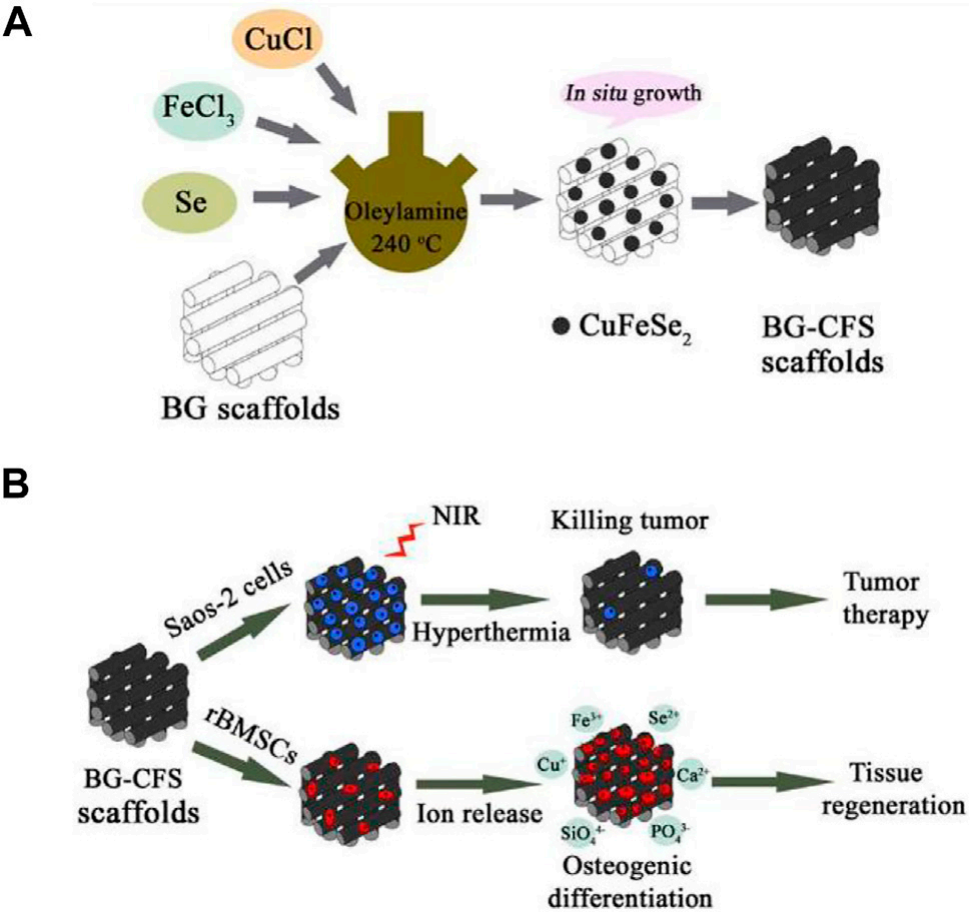
Illustration showing **(A)** CuFeSe2 nanocrystals on BG scaffolds and **(B)** their bifunction of bone tumor therapy and tissue regeneration. Reproduced with permission from (Dang et al., 2018).

CuFeSe2 nanocrystals may develop *in situ* on the strut surface of BG SCF using the solvothermal reaction technique, resulting in tight interactions between CuFeSe2 nanocrystals and BG SCF (Figure 6). The narrow energy band of CuFeSe2 (0.16 eV) was attributed to the good photothermal effect of bifunctional SCF, leading in effective light absorption. The released Ca, Si, P, Fe, Cu, and Se ions may work synergistically to induce rabbit bone MSC osteogenesis. A new bifunctional scaffolding containing CuFeSe2 nanocrystals might be used as a therapeutic treatment approach for tumor-induced bone abnormalities (Dang et al., 2018).

Elements doped BG-ceramic (BGC) SCF demonstrated photothermal effect and osteogenic differentiation capacity utilizing the 3DP technique. The photothermal effect followed this pattern: 5Cu-BGC is superior. The produced high temperature was efficient in killing tumour cells and inhibiting tumor growth *in vivo* due to the photothermal impact of 5Cu-BGC, 5Fe-BGC, and 5Mn-BGC SCF. Furthermore, the ionic products produced by element-doped SCF appeared to stimulate the osteogenic differentiation of bone-forming cells, and 5Fe-BGC and 5Mn-BGC SCF enhanced the affection of rabbit bone MSCs. The effect of 5Co-BGC SCF on ALP expression was substantially greater than the effect of other SCF. Furthermore, as compared to a blank control, element-doped SCF clearly enhanced VEGF expression in rabbit bone MSCs. According to the findings, 5Fe-BGC and 5Mn-BGC SCF might be used in the photothermal therapy of bone tumors and BTR (Liu et al., 2018).

Synergy aimed photothermal and reactive oxygen species (ROS) treatments outperformed solo photothermal therapy in terms of tumor therapeutic efficacy. 3DP was used by Wu et al. to construct CaSiO3-Fe complex SCF (Liu et al., 2018). The complex SCF had a great tensile grift, which meant they could sustain bone cortical defects mechanically. Furthermore, *in vitro* and *in vivo*, the photothermal impact of Fe and the Fenton reaction between the liberated Fe ions and H_2_O_2_ in tumor cells led in synergistic photothermal and ROS treatments for tumors. *In vivo*, the inclusion of CaSiO3 in composite SCF increased the rate of breakdown, enhanced rBMSC proliferation and differentiation, and encouraged bone formation. Such high-compressive-strength composite SCF can be used as adaptable biomaterials for the repair of cortical bone defects and the treatment of bone tumors.

**FIGURE 7:**
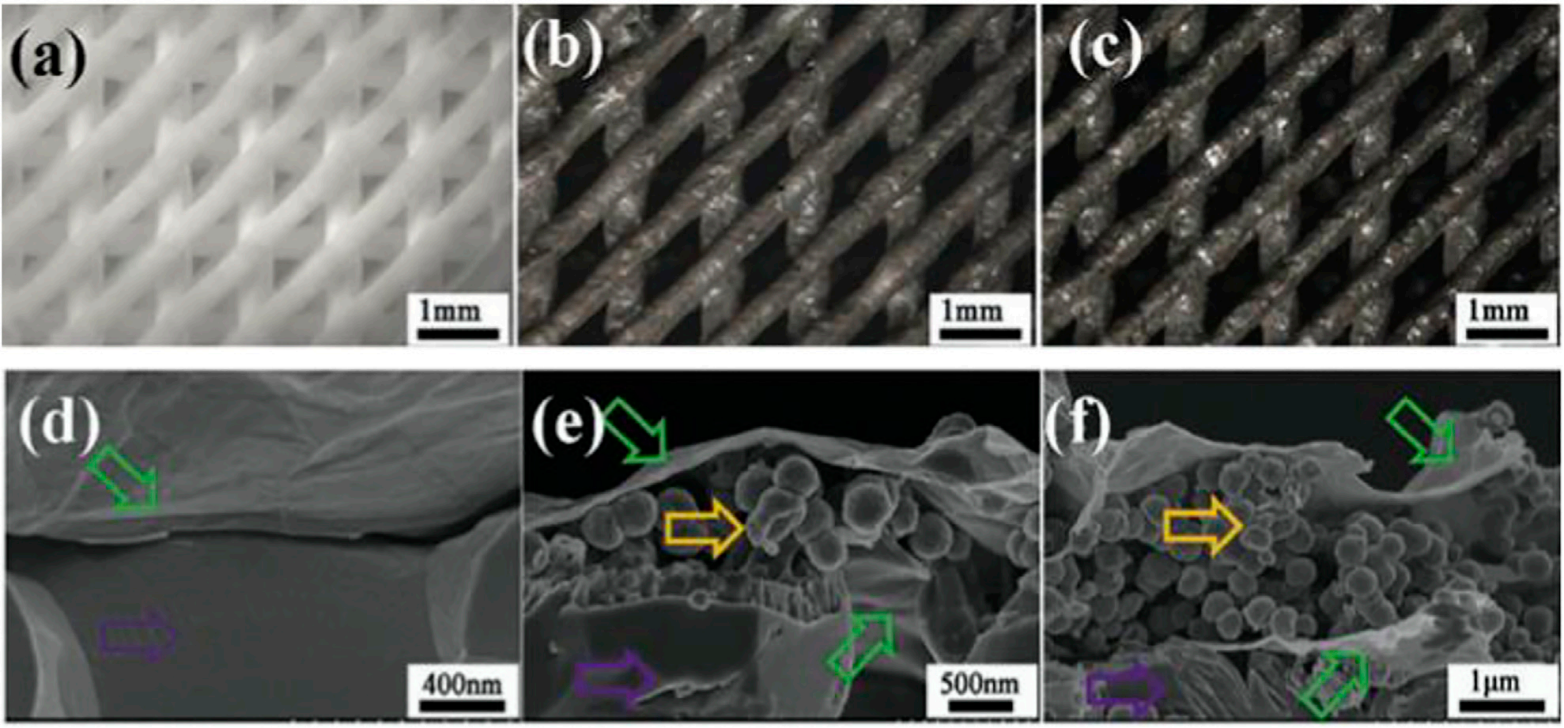
Optical micrographs of b-TCP, b-TCP–4Fe–GO, and b-TCP–8Fe–GO scaffolds **(A)**, b-TCP–4Fe–GO **(B)**, and b-TCP–8Fe–GO scaffolds **(C)**. The fracture surfaces of b-TCP–GO **(D)**, b-TCP–4Fe–GO **(E)**, and b-TCP–8Fe–GO **(F)** scaffolds revealed sandwich-like layers in the magnetic scaffolds.

Magnetothermal treatment, photothermal therapy, can be used to destroy tumor cells using functional magnetic SCF. Because of the success of creating superparamagnetic SCF, an oscillating magnetic field can be used to destroy remaining tumor cells locally. Zhang et al. created a magnetic scaffold using a GO-Fe3O4-GO sandwich layer. With only a few Fe3O4 particles on the surface of the SCF, this sandwich layer had a better magnetothermal effect (Figure 7). Furthermore, the presence of GO is advantageous for heat transmission due to its high thermal conductivity, and for stimulating new bone production (Zhang et al., 2016).

Patients may develop bacterial infections when using implanted materials, posing a significant danger. Zhang et al. created silver (Ag)/GO particle-modified b-TCP BCR SCF called Ag@GO. The inclusion of Ag ion in the SCF resulted in significant antibacterial activity. GO has a number of beneficial characteristics, including conductivity, biocompatibility, and excellent mechanical properties. They focused on its capacity to boost BMSC angiogenesis and mass formation in this investigation (Zhang et al., 2017c).

## Conclusion

“In conclusion, when it comes to realizing extraordinary organizational complication, adaptability, and patient-specific needs, 3DP technology clearly outperforms older approaches. In terms of content and hierarchical structure, this study detailed the progress of 3DP-BSCF for presentations ranges from BTE to bone cancer management (macros, micros, and nanos levels). We focused on the impact of scaffold composition and hierarchical structure on physical, chemical, and biological characteristics. 3DP SCF have a hierarchical structure that can provide anchoring sites for cell expansions, promote cell spread, and further govern cell network development and tissue ingrowth. We described certain bi-functional BCR SCF in particular that have good photothermal or antibacterial performance while also promoting bone tissue regeneration. Such bi-functional BCR SCF provide up new avenues for the treatment of bone tumors. Surely, the current 3DP technology has certain drawbacks in terms of additional needs. Processability and regenerability are critical in tissue engineering, and factors including mechanical properties, degradation rate, and scaffold precision must be continually improved. As a result, 3DP technology’s precision and digital operating system must be continually enhanced. In the future, better spatial resolution than the current resolution given by main 3DP methods will be required to create 3DP ceramic SCF that mimic the natural macros, micros, and nanos structures of the bone.”

“To avoid powdered particle aggregation, one option is to utilize powdered granules with excellent flowability and an appropriate binder solution. Furthermore, as an emerging technology, a high-performance 3DP will be costly. To cut costs, greater technical innovation and multidisciplinary collaboration are required. 3DP SCF for BTR still require post-treatments like high-temperature sintering or densification. The shrinking of various portions of the SCF during the sintering process may be uneven, causing the SCF to fracture and become useless. The micropore and microstructure of 3DP SCF generated by 3DP cannot be smaller than 10 lm due to resolution limitations. Nanomaterials are integrated into 3DP inks or changed on the surface of 3DP-BSCF to create microstructures. Furthermore, the sintering procedure has a significant impact on the BCR SCF′ microporosity. During the sintering process, the binder will be removed from the SCF, revealing the micropores. The microporosity of sintered SCF is determined by the binder concentration. In the case of bone tumour therapy, a compelling big animal model is required to test the bi-functional BCR SCF’ potential to be utilized for both bone tumor therapy and BTR in the same animal. The following are thought to be the key issues and obstacles in the development and implementation of 3DP biological tissue-engineered SCF in hospital: 1) the difficulties in controlling the accuracy of 3DP bioactive SCF based on medical individualization needs; 2) the vast differences in mechanical properties between 3DP-B SCF and the clinical requirements of implanted materials under various indication conditions; 3) the difficulty in repairing large bone defects with 3D-printed bioactive SCF under various indication conditions”.

“Biological 3DP technology is a novel approach that has just been introduced to the 3DP sector. This new area, which entails printing tissue-engineered SCF that sustain living cells and even live organs, has a lot of potential. The greatest danger, however, is that the cell structure will be badly disrupted during the printing process. As a result, the major challenge to address during material preparation is maintaining the integrity and activity of the cells before and after printing. It will also be a significant opportunity and challenge to use biological 3DP technology to print certain bi-functional BCR SCF with outstanding photothermal or osteogenic concert. Although current printing methods may generate SCF with structures that are comparable to those of many tissues, completely functional tissue engineered SCF are still a long way off. 3DP tools is an influential inundation that would persist to propel different areas forward. 3DP bioactive SCF will be a great fit for bone abnormalities in the future, and will fulfil clinical individualization criteria. 3DP will be increasingly precise and low-cost as a result of multidisciplinary collaboration and technical progress. Furthermore, some of them will be quite small and portable, and our electronic equipment will be able to manage and reassemble them”.
